# Adult Smokers’ Awareness and Interest in Trying Heated Tobacco Products: Perspectives from Mexico, where HTPs and E-Cigarettes are Banned

**DOI:** 10.3390/ijerph17072173

**Published:** 2020-03-25

**Authors:** Lizeth Cruz-Jiménez, Inti Barrientos-Gutiérrez, Liliana Coutiño-Escamilla, Katia Gallegos-Carrillo, Edna Arillo-Santillán, James F. Thrasher

**Affiliations:** 1 Evaluation and Surveys Research Center, National Institute of Public Health, Cuernavaca, Mor 62100, Mexico; inti.barrientos@insp.mx; 2 Tobacco Research Department, National Institute of Public Health, Cuernavaca, Mor 62100, Mexico; lcoutinoe@gmail.com (L.C.-E.); katiagal@usc.edu (K.G.-C.); edna@insp.mx (E.A.-S.); 3 Epidemiology and Health Services Research Unit, Mexican Institute of Social Security, Cuernavaca, Mor 62000, Mexico; 4 School of Demography, Australian National University, Canberra 0200, Australia; 5 Department of Health Promotion, Education & Behavior, Arnold School of Public Health, University of South Carolina, Columbia, SC 29208, USA

**Keywords:** heated tobacco product, nicotine, ENDs/ANDs, LMICs

## Abstract

Background: We evaluated smokers’ perceptions of heated tobacco products (HTPs) in Mexico, where industry publically lobbied to introduce HTPs into this country that banned both HTPs and e-cigarettes. Methods: Online surveys (November 2018 to July 2019) were analyzed from adults who only smoked cigarettes (*n* = 2091) or who smoked and used e-cigarettes (“dual users” *n* = 1128). Logistic models regressed HTP awareness, interest to trying HTPs, and having seen HTPs for sale (only among aware participants) on sociodemographics and tobacco-related variables. Results: Of the 17.1% who were aware of HTPs, 52.7% reported having seen HTPs for sale. Of all respondents, 75% were somewhat or very interested in trying HTPs. Compared to their counterparts, more frequent smokers, dual users, those exposed to online e-cigarette ads, and those with friends who used e-cigarettes were both more aware of and interested in trying HTPs. Greater awareness was also associated with higher education, recent attempts to quit, receipt of email e-cigarette ads, and smoking among friends and family. Seeing HTPs for sale was higher for those who recently attempted to quit, were exposed to e-cigarette ads online or by email, or had friends who used e-cigarettes. Conclusion: Interest in HTPs is high among smokers in Mexico, which already has a large black market for illegal e-cigarettes. HTPs use should be monitored in this context, especially given the public health impacts of HTPs are unclear.

## 1. Introduction

Heated tobacco products (HTPs) deliver nicotine aerosols to consumers by heating tobacco [1,2], potentially reducing exposure to harmful chemicals that result from burning tobacco, as with cigarettes. In recent years, Philip Morris International (PMI) has been at the forefront of innovating and promoting HTPs around the world, particularly their HTP product IQOS (‘I Quit Ordinary Smoking’). Since its launch in 2014 in Japan and in 2015 in Switzerland and Italy [2], IQOS has rapidly expanded to markets in 45 countries, accompanied by industry projections of strong market growth [3,4]. Consumers’ perceptions of HTPs have been studied in high-income countries, including those where HTPs have been introduced [5,6,7,8,9]. However, these studies do not characterize the profiles of smokers who are aware of HTPs and could be interested in testing them. Furthermore, no studies of HTPs have been conducted in low- or middle-income countries, which increasingly bear the burden of tobacco-related disease and where industry is beginning to market HTPs. In Mexico, 2008 tobacco legislation prohibits the marketing of novel nicotine-containing products, such as e-cigarettes and HTPs. In this legislative context, e-cigarettes are primarily obtained through social networks, online, and informal economic channels [10]. Whether Mexican consumers can access HTPs has not been studied, but since late 2018, PMI has publically campaigned to enter the legal market. The present study assesses the level and correlates of adult smokers’ awareness of and interest in HTPs in this context. 

The tobacco industry represents HTPs as less harmful than cigarettes, arguing that heating tobacco produces an aerosol with lower levels of the carbon monoxide (CO), tar, and carcinogenic compounds than levels in the smoke from combusted cigarettes [11]. However, many have questioned the reduced risk claims of HTPs [12], pointing out that HTPs still produce a significant amount of dangerous tobacco-specific nitrosamines (TSNA) [13]; that there is little difference between HTPs and cigarettes in the total gaseous and particulate compounds [14]; no meaningful differences from smoking cigarettes for most biomarkers of potential harm [15]; that HTPs may lead to lower risk of some smoking-caused diseases but may result in higher risk for other diseases [1,16]; and that even if their risk is lower than for cigarettes, it is still higher than for e-cigarettes [17]. Some countries, such as the USA, have allowed HTPs onto the market, although the US Food and Drug Administration has not yet decided whether it will authorize their marketing as a reduced risk product [18]. Nevertheless, the rapid growth of the HTP market in some countries, such as Japan and South Korea [19], is indicative of the potential appeal of these products for smokers. Unfortunately, information about HTPs is, at present, largely limited to industry reports. Research independent of the industry is required to better inform conclusions about the potential public health impact of this product.

Studies in high-income countries where PMI has introduced IQOS have found that awareness of and interest in using HTPs were higher among youth, males, smokers, and e-cigarette users [5,6,7,8,9]. No consistent results have been found for correlations such as age [5,6,7,8,9], education, [6,9] or income [6], although the adoption of new technologies is generally more frequent amongst younger consumers and those from higher socioeconomic status groups [20]. Although the current evidence on the appeal of HTPs is limited, other potential correlates are worth exploration. For example, HTPs appeal to smokers who embrace other cigarette product innovations, such as flavor capsules in the filter that consumers can crush to flavor the smoke, which are particularly popular in Latin America [21,22]. Indeed, in some countries, industry markets HTPs with flavor capsules [23]. In addition, other studies have found positive correlations between HTP use and alcohol consumption [24,25], drug use [25], intentions to quit smoking, and advertising exposure [24]. No studies of which we are aware have examined the characteristics of adult smokers who are more aware of or interested in trying HTPs in low-and middle-income countries. Nevertheless, we evaluate whether the correlations found in high-income countries generalize to the Mexican context.

### Study Context

**Figure 1:**
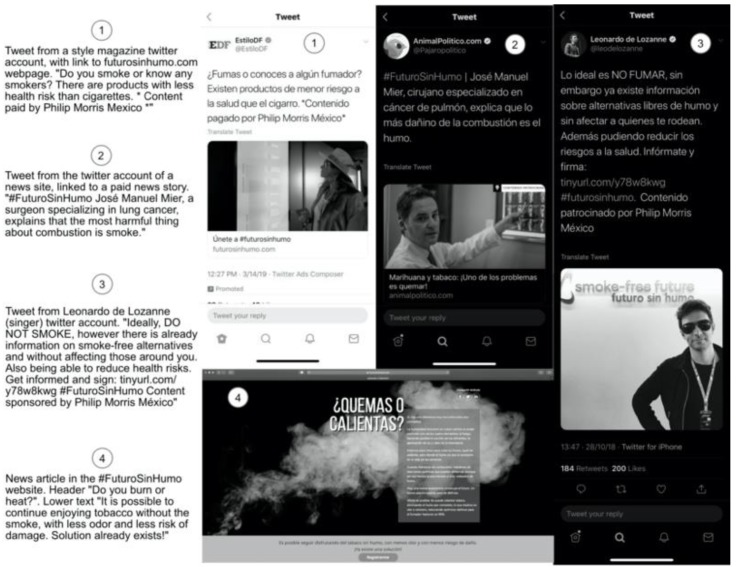
Examples of promotional materials use in Mexican social media.

In Mexico, 17.5% or 14.9 million of those aged 12 to 65 are current smokers. Smokeless tobacco use is low (0.6%). While only 1.1% currently used e-cigarettes in 2016 [26], use has been growing and was much higher among smokers (5%), particularly young adult smokers (9%) [10]. The General Law for Tobacco Control in Mexico bans the distribution, marketing, and sales of products that look or function like cigarettes, which regulators have interpreted to include e-cigarettes. HTP’s, in theory, could comply with the requirements and limitations set forth in the law, but regulatory agencies had not granted permission for marketing HTPs [27]. However, since 2018, PMI has promoted IQOS through lobbying with legislators and stakeholders, social media campaigns (#FuturoSinHumo), paid news stories in newspapers and magazines, and a website (https://futurosinhumo.com). Furthermore, IQOS online and social media presence in other countries is easily accessible in Mexico. As in other countries, IQOS is marketed by PMI as a sophisticated, high tech device that provides all the sensations of smoking cigarettes but with less ash and odor [28]. Health claims are both direct and implicit in their campaign messages (see Figure 1) that an IQOS is ”smoke free”, ”doesn’t affect the people around you”, and ”reduces health risks”, potentially generating the perception that HTPs are less risky than other tobacco products [29]. It is not known whether the PMI’s campaign generated consumer awareness or interest in trying IQOS in Mexico, where legal HTPs could have a competitive advantage over e-cigarettes, whose marketing and sales are banned, similar to most other Latin American countries [30]. Despite the illegality of e-cigarettes, use is relatively common among Mexican adolescents [31,32] and adult smokers [10], who also find legal HTPs appealing. Thus, this study aimed to identify the prevalence and correlates of awareness and interest in trying HTPs among Mexican adult smokers, including those who use e-cigarettes. We expect to find a relatively low level of awareness and of having seen HTPs for sale, given that HTPs are illegal, whereas they are legal in other countries where they have been studied. However, the correlates of interest in trying HTPs are likely to be similar to those found in other countries, including being as high as other attractive and alternative nicotine products. The three independent variables tested should correlate with at least one, smoking-related variable, as reported in the previous literature.

## 2. Methods 

### 2.1. Data Source

Data were analyzed from three waves of an online survey of Mexican adult smokers or e-cigarette users recruited from a consumer panel for marketing research. Participants had to be over 18 years of age and have smoked or used e-cigarettes in the prior month. Surveys were administered among approximately 1500 participants every four months (November 2018, March 2019, and July 2019), with some participants followed over time and new participants recruited to replenish and maintain the same sample size at each wave. Surveys were administered in Spanish using questions from the International Tobacco Control (ITC) survey [33] and other surveys of new tobacco product use in Mexico [31,32], for which gold standard committee translation methods and pretesting with cognitive interviews had been conducted. The survey took between 20 and 25 min to complete, on average, and the survey company provided a standard incentive for participation. All study procedures were approved by the Institutional Review Board and Ethics Committee of the National Institute of Public Health of Mexico (Ethical Approval Code: CI 1572). The present study includes only data from the first survey to which participants responded (Wave 1 *n* = 1501, Wave 2 *n* = 1035, and Wave 3 *n* = 799), in order to ensure that prior survey participation (i.e., answering questions about HTPs) did not influence responses. In other words, we did not include data from subsequent waves for those who were successfully followed up.

### 2.2. Measures

#### 2.2.1. Dependent Variables

**Figure 2:**
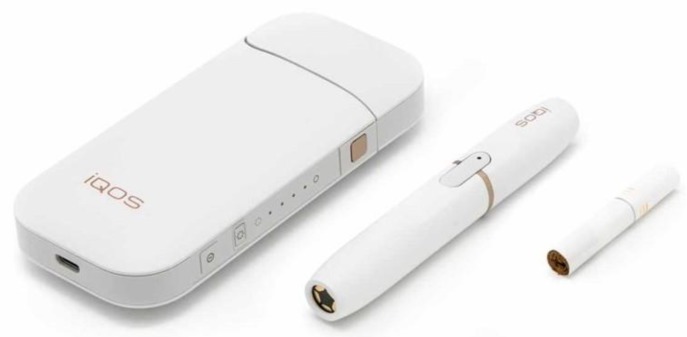
Picture and description of IQOS provided to respondents.

After providing a description of HTPs and a photo of IQOS (See Figure 2), the three dependent variables were assessed as follows: (1) awareness (i.e., “Have you heard about new electronic products that heat tobacco instead of burning it?”no = 0 and yes = 1); (2) those who indicated awareness were asked if they had seen HTPs for sale (“Have you ever seen any of these heat-not-burn products for sale in a store or online in Mexico?” no = 0 and yes = 1); (3) interest in trying HTPs (“Would you be interested in trying one of these heat-not-burn products if you had the opportunity?”) with response options dichotomized (i.e., “not at all” or “a little interested” = 0; “somewhat interested” and “very interested” = 1).

#### 2.2.2. Independent Variables

The independent variables included those related to smoking-related variables as follows: smoking frequency (i.e., non-daily, daily ≤5 cigarettes/day, and daily >5 cigarettes/day); vaping frequency (i.e., exclusive smoker; dual user, sporadic use of e-cigarettes, less than once a week; dual user, frequent use of e-cigarettes, at least once a week); preference for cigarette brands with flavor capsules (i.e., no/yes); recent attempt to quit smoking (i.e., in the prior 4 months); and quit intentions (“sometime in the future, after 6 months”, “not at all”, and “don’t know” = 0; “in the next month” and “in the next six months” = 1). In addition, participants were asked if, in the prior 30 days, they had seen any advertising for e-cigarette devices or e-liquids online (i.e., websites or social media sites, no/yes) or by email/text message (i.e., no/yes). Participants also reported their consumption of other substances, including binge drinking (“How often do you have 6 or more drinks on one occasion?”) with response options dichotomized (i.e., “never”, “less than monthly”, and “monthly” = 0; “weekly” and “daily or almost daily” = 1). Marijuana use in the last month was classified as none, once, and more than once. Participants reported smoking and e-cigarette use among their close friends with whom they regularly spent time, as well as among househdold members, with responses dichotomized as follows: friends smoke (no/yes), friends use e-cigarettes (no/yes), family smokes (no/yes), and family uses e-cigarettes (no/yes).

#### 2.2.3. Covariates

Other variables evaluated included: gender (female/male), age (18–29, 30–39, 40–49, and >50 years), education (less than high school, high school graduate, some college, and college degree or higher), household income (less than 8000 MXN monthly; 8001 to 15,000 MXN monthly; 15,001 to 20,000 MXN monthly; >20,000 at monthly; and do not know) and survey wave (Wave 1, November 2018 = reference; Wave 2, March 2019; and Wave 3, July 2019).

### 2.3. Analysis

We evaluated the descriptive statistics for all variables of interest. Separate independent logistic regression models were estimated for each outcome at a significance level of *p* < 0.05 for awareness, seen for sale (in the subsample of those who were aware of HTPs), and interest to trying IQOS. All models included smoking-related variables, sociodemographic characteristics, and survey wave. In addition, we re-estimated the model predicting interest by analyzing only the subsample of those who indicated prior awareness of HTPs. The results were consistent with those from the full model for key variables (e.g., higher interest was found among those who recently attempted to quit smoking, who were recently exposed to e-cigarette ads online, and who use e-cigarettes more frequently (see Supplementary Table S1). We only reported the results from models with the entire sample because we believe that it is important to understand the potential appeal of HTPs for all smokers given that HTPs would be made available to all if they were to come onto the market. Analyses were conducted using Stata v.14 (StataCorp, Lakeway Dr, College Station, TX, USA). 

## 3. Results

The sample (*n* = 3219) was half (51.3%) male and mostly under age 39 (18 to 29 years old = 35.1% and 30 to 39 years old = 30.6%). About a third of participants had an educational attainment of high school or less (36.0%) and another third (33.9%) had a college degree or higher. A third of the sample vaped in the prior month, and most of them used e-cigarettes less than once a week. Almost half (45.8%) of respondents participated in the first wave of data collection (Table 1).

### 3.1. Factors Associated with Being Aware of HTPs

The prevalence of HTPs awareness was 17.1% (Table 1). Awareness was highest among the 30 to 39 years old (23%) and among those with a college degree or higher (24%), and these differences remained statistically significant in adjusted models (See Table 2). A comparison with exclusive smokers showed that awareness was higher amongst dual users, whether they used e-cigarettes sporadically (AOR = 1.60, 95% CI 1.22 to 2.10) or more frequently (AOR = 1.99, 95% CI 1.48 to 2.69). More frequent smoking was also associated with higher awareness (AOR daily 5+ cpd vs. non-daily = 1.31, 95% CI 1.00 to 1.69) and who reported a recent quit attempt (AOR = 1.43, 95% CI 1.15 to 1.81). Other correlates of higher awareness included online ad exposure (26% vs. 9% respectively; AOR = 1.64, 95% CI 1.30 to 2.06) receipt of e-cigarette ads by email (AOR = 2.93, 95% CI 2.29 to 3.74), and marijuana use more than once in the last month (AOR = 1.34, 95% CI 1.01 to 1.78). Finally, tobacco product use among friends and family were significantly associated with greater awareness of HTPs.

### 3.2. Seeing HTPs for Sale

Among respondents who reported being aware of HTPs (17% of the analytical sample), 52.7% reported seeing HTPs for sale online in Mexico. Reported sales were higher for those who also reported exposure to e-cigarette ads, either online (60% vs. 35%, AOR = 1.98, 95% CI 1.32 to 3.11) or by email (69% vs. 43%, AOR = 1.90, 95% CI 1.23 to 2.92). Seeing HTPs for sale was also higher among those who reported a recent attempt to quit smoking (59% vs. 46%, AOR = 1.80, 95% CI 1.17 to 2.77) and plan to quit in the next six months (52% vs. 53%, AOR = 0.57, 95% CI 0.37 to 0.89). Other factors associated with reported sales of HTPs were marijuana use more than once in the last month (72% vs. 44% among those who did not use marijuana in the last month, AOR = 1.87, 95% CI 1.11 to 3.15). Finally, e-cigarette use among friends (AOR = 1.83, 95% CI 1.11 to 3.01, Table 3) were associated with seeing HTPs for sale. To evaluate why plans to quit became statistically significant in adjusted models, we re-estimated models after removing each of the covariates, one at a time, then returning the variable to the model before removing the next variable and re-estimating the model again. As we suspected, the recent quit attempt variable explains this unexpected result due to its correlation with plans to quit.

### 3.3. Factors Associated with the Interest in Trying HTPs

Overall, interest in trying HTPs was high (75%), but it was significantly higher among those with the greatest household income (i.e., more to 20,000 Mexican pesos a month) as compared with those with the lowest household income (i.e., less than 8000 pesos, AOR = 1.37, 95% CI 1.05 to 1.79, see Table 4). Compared to exclusive smokers, dual sporadic users (80% vs. 71%, respectively, AOR = 1.42, 95% CI 1.10 to1.82) and dual frequent users (87% vs. 71%, respectively, AOR = 1.90, 95% CI 1.37 to 2.64) were more interested in trying HTPs. In addition, compared with non-daily smokers, interest was higher amongst those who smoked five or fewer cigarettes per day (77% vs. 71%, respectively, AOR = 1.41, 95% CI 1.13 to 1.74) or more than five cigarettes per day (81% vs. 71%, respectively, AOR = 1.79, 95% CI 1.42 to 2.25). Other independent correlates included recent attempt to quit smoking (77% vs. 73%, AOR = 1.22, 95% CI 1.01 to 1.48), online ads exposure (AOR = 1.77, 95% CI 1.47 to 2.15), binge drinking (82% vs. 73%, respectively, AOR = 1.54, 95% CI 1.24 to 1.92) marijuana use in the last month (70% vs. 75%, respectively, AOR = 0.65, 95% CI 0.50 to 0.86), and e-cigarette use among close friends (82% vs. 71%, AOR = 1.35, 95% CI 1.08 to 1.68).

## 4. Discussion

To the best of our knowledge, this is the first study to evaluate the prevalence of and factors associated with awareness or interest in trying HTPs in low- or middle-income countries. We found that a minority of Mexican smokers and dual users (17.1%) were aware of HTPs, but this was higher than reported for adult smokers in the USA (10%) in 2017 [8], when HTPs were not yet allowed on the US market. Almost three years after PMI’s IQOS was launched in Italy, awareness among current smokers was somewhat higher (26.4%) [34]. Surprisingly for our study, about half of smokers who were aware of HTPs (52.7%) reported having seen HTPs for sale online or in Mexico in spite of HTP sales being banned at the time of the surveys. This estimate could over-represent HTP access, since some participants could have misperceived as “sales” some of the media coverage and other industry efforts to promote awareness. At the same time, however, having really seen illegal HTPs for sale is not too far fetched given the extent of contraband e-cigarette consumption in Mexico, including in this online sample where one third had used e-cigarettes in the prior month.

Consistent with prior research in Italy, [34] middle-aged adults (30–39 age) were most likely to be aware of HTPs. Marketing strategies for HTPs in other countries have been found to appeal to the nostalgia for combustible products (taste and behavioral process), along with aspirational ideas that also could appeal to younger adults and adolescents [28]. As such, it is noteworthy that awareness was higher among middle-aged than young adult smokers. Indeed, HTPs could appeal more to middle-aged adults because of their longer histories of smoking cigarettes, which are more similar to HTPs than to e-cigarettes that are particularly popular among younger adults and adolescents [10].

The present study was carried out when no HTP was legally on the market. The fact that exposure to online advertising was associated with awareness and interest in trying HTPs is not too surprising given that such exposures are also associated with e-cigarette susceptibility [35], which is also illegal in Mexico. Indeed, internet marketing is difficult to regulate, including because of access to internationally transmitted content. Furthermore, large social media campaigns have accompanied the launch of IQOS [28], which could have resulted in further online information sharing and seeking [36] and boosting of ideas to mislead consumers about HTPs safety, especially when the evidence is inconclusive about it [37,38,39,40]. As IQOS and other HTPs enter and are marketed in Mexico, awareness and use will certainly increase, as it has in other countries [5,6,24], yet market growth for this tobacco product segment needs to be studied for Mexico, where smoking frequency is significantly lower than in other populations around the world [31,32,41].

Consistent with a recent study [25], use of e-cigarettes, marijuana, and alcohol were all associated with greater awareness of HTPs and interest in trying them, which suggests that clustering of substance use likely expands to encompass new substances such as HTPs. Similar to the established social influences on other tobacco products, such as e-cigarette initiation and maintenance [35,42], we found that e-cigarette use amongst friends was positively associated with all outcomes studied. Due to homophily amongst close friends, having network members who use e-cigarettes could indicate a general openness to other novel technologies such as HTPs, independent of one’s own e-cigarette use behavior. Sharing information about new products could not only lead to awareness, but also opportunities to purchase these products [43].

The study results presented here are limited. The sample was recruited from a convenience sample that has been purposefully selected to represent key market segments in Mexico; however, the sample is not representative of the Mexican general population of smokers, as we over-represented younger smokers, those from higher SES groups, and smokers who also use e-cigarettes [10]. As such, we likely overestimated the prevalence of each outcome studied; nevertheless, this population is also likely to include those most open to adoption of new products such as HTPs. Hence, this population of potential “early adopters” provides important insights into the initial uptake of HTPs, and our results suggest there will be substantial interest. Be that as it may, the results of this study suggest that direction of future research is to explore awareness and interest in HTPs for nonsmokers and youth, issues of continuing concern, especially when prior studies have suggested that these products appeal to never smokers and serve as a gateway to nicotine addiction [6,34,44]. In this sense, prior studies have reported that nearly half of Italian IQOS users and over half of the people interested in IQOS are never smokers [9,34]. There is also a need the further exploration of the perceptions and other characteristics associated with awareness and the interest to try HTPs.

## 5. Conclusions

Our study suggests that current smokers and dual users are mostly interested in trying HTPs. Since October 2019, PMI’s IQOS has been on sale in Mexico, although regulatory agencies had not granted permission for marketing HTPs. Monitoring HTPs use and its consequences is of the utmost importance for informing tobacco regulations, especially in countries where the laws are not clear or do not exist. The findings would contribute surveillance to help stakeholders prepare appropriate regulations for HTPs.

## Figures and Tables

**Table 1:** Sample characteristics (*n* = 3219).

Variable	%	*n*
Awareness of HTPs	17.1	550
Seeing HTPs for sale *	52.7	290
Interest in trying HTPs	75.0	2415
**Gender**		
Female	48.7	1568
Male	51.3	1651
**Age**		
18–29	35.1	1130
30–39	30.6	984
40–49	17.6	565
>50	16.8	540
**Education**		
Less than high school	9.2	295
High school graduate	36.0	1159
Some college	20.9	673
College degree or higher	33.9	1092
**Household income**		
Less than 8000 MXN monthly	23.7	762
8001 to 15,000 MXN monthly	28.5	917
15,001 to 20,000 MXN monthly	17.0	546
>20,000 MXN at monthly	25.9	835
Don’t know	4.9	159
**Data collection**		
Wave 1	45.8	1473
Wave 2	30.9	995
Wave 3	23.3	751
**Tobacco product use**		
Exclusive conventional smoker	65.0	2091
Dual sporadic user	21.8	701
Dual frequent user	13.3	427
**Cigarette consumption**		
Not daily	53.8	1731
Daily ≤ 5 cigarettes	21.8	701
Daily > 5 cigarettes	24.5	787
**Flavor capsule use**		
No	40.1	1290
Yes	59.9	1929
**Smoking quit attempt**		
No	57.0	1835
Yes	42.9	1384
**Plan to quit**		
Sometime in the future	62.4	2010
In the next six months	37.6	1209
**Tobacco ads on Internet**		
No	53.8	1732
Yes	46.2	1487
**Tobacco ads by email**		
No	85.5	2751
Yes	14.5	468
**Binge drinking**		
No	76.8	2471
Yes	23.2	748
**Marijuana use in the last month**		
None	77.0	2478
Once	10.3	333
More than once	12.7	408
**Friends smokes**		
No	16.1	518
Yes	83.9	2701
**Friends use e-cigarettes**		
No	62.2	2003
Yes	37.8	1216
**Family smokes**		
No	38.9	1252
Yes	61.1	1967
**Family uses e-cigarettes**		
No	77.0	2479
Yes	23.0	740

* The 100% correspond to *n* = 550.

**Table 2:** Factors associated with the awareness of IQOS (‘I Quit Ordinary Smoking’) among adult smokers (*n* = 3219).

Variable	%	Univariate Estimate		Adjusted Estimates	
		OR	(95% CI)	AOR	(95% CI)
**Gender**					
Female	16	Ref		Ref	
Male	18	**1.20 ^1^**	(1.00–1.45)	1.12	(0.91–1.39)
**Age**					
18–29	17	Ref		Ref	
30–39	23	**1.48 ^3^**	(1.20–1.84)	**1.40 ^2^**	(1.09–1.80)
40–49	14	0.80	(0.61–1.08)	1.00	(0.72–1.40)
>50	10	**0.58 ^2^**	(0.42–0.79)	1.18	(0.81–1.72)
**Education**					
Less than high school	8	Ref		Ref	
High school graduate	14	**1.82 ^2^**	(1.16–2.86)	**1.63 ^1^**	(1.00–2.65)
Some college	15	**1.92 ^2^**	(1.20–3.08)	1.45	(0.87–2.43)
College degree or higher	24	**3.65 ^3^**	(2.35–5.67)	**1.85 ^1^**	(1.12–3.07)
**Household income**					
Less than 8000 MXN monthly	15	Ref		Ref	
8001 to 15,000 MXN monthly	14	0.96	(0.73–1.25)	0.75	(0.55–1.02)
15,001 to 20,000 MXN monthly	20	1.45	(1.09–1.93)	1.05	(0.75–1.47)
>20,000 MXN monthly	22	**1.62 ^3^**	(1.25–2.09)	1.05	(0.76–1.46)
Don’t know	5	**0.30 ^3^**	(0.14–0.63)	**0.46 ^1^**	(0.21–0.99)
**Data collection**					
Wave 1	19	Ref		Ref	
Wave 2	15	**0.82 ^2^**	(0.65–1.00)	0.95	(0.75–1.21)
Wave 3	16	0.86	(0.68–1.09)	0.90	(0.69–1.17)
**Tobacco product use**					
Exclusive conventional smoker	10	Ref		Ref	
Dual sporadic user	29	**3.68 ^3^**	(2.96–4.57)	**1.60 ^2^**	(1.22–2.10)
Dual frequent user	33	**4.61 ^3^**	(3.60–5.90)	**1.99 ^3^**	(1.48–2.69)
**Cigarette consumption**					
Non-daily	15	Ref		Ref	
Daily ≤ 5 cigarettes	17	1.16	(0.92–1.47)	1.12	(0.86–1.46)
Daily > 5 cigarettes	21	**1.49 ^3^**	(1.20–1.85)	**1.31 ^1^**	(1.00–1.69)
**Flavor capsule use**					
No	14	Ref		Ref	
Yes	19	**1.54 ^3^**	(1.26–1.87)	0.99	(0.80–1.25)
**Recent quit attempt**					
No	14	Ref		Ref	
Yes	22	**1.75 ^3^**	(1.46–2.11)	**1.43 ^2^**	(1.15–1.81)
**Plan to quit**					
Sometime in the future	15	Ref		Ref	
In the next six months	20	**1.42 ^3^**	(1.18–1.71)	1.01	(0.81–1.28)
**E-cigarette ads on Internet**					
No	9	Ref		Ref	
Yes	26	**3.35 ^3^**	(2.75–4.09)	**1.64 ^3^**	(1.30–2.06)
**E-cigarette ads by email**					
No	12	Ref		Ref	
Yes	44	**5.64 ^3^**	(4.55–6.98)	**2.93 ^3^**	(2.29–3.74)
**Binge drinking**					
No	16	Ref		Ref	
Yes	22	**1.57 ^3^**	(1.28–1.92)	1.01	(0.80–1.28)
**Marijuana use in the last month**					
None	14	Ref		Ref	
Once	26	**2.12 ^3^**	(1.62–2.78)	1.28	(0.93–1.75)
More than once	29	**2.58 ^3^**	(2.02–3.28)	**1.34 ^1^**	(1.01–1.78)
**Friends smokes**					
No	9	Ref		Ref	
Yes	19	**2.42 ^3^**	(1.75–3.33)	**1.49 ^1^**	(1.05–2.14)
**Friends use e-cigarettes**					
No	10	Ref		Ref	
Yes	29	**3.94 ^3^**	(3.2–4.77)	**1.57 ^3^**	(1.22–2.01)
**Family smokes**					
No	12	Ref		Ref	
Yes	20	**1.84 ^3^**	(1.50–2.25)	**1.33 ^1^**	(1.04–1.69)
**Family uses e-cigarettes**					
No	13	Ref		Ref	
Yes	32	**3.17 ^3^**	(2.60–3.85)	1.28	(0.99–1.66)

Significant values in bold: ^1^ *p*-value, *p* < 0.05; ^2^ *p*-value, *p* < 0.01; ^3^ *p*-value, *p* < 0.001.

**Table 3:** Factors associated with having seen heated tobacco products (HTPs) for sale, among adult smokers who were aware of HTPs (*n* = 550).

Variable	%	Univariate Estimates		Adjusted Estimates	
		OR	(95% CI)	AOR	(95% CI)
**Gender**					
Female	52	Ref		Ref	
Male	53	1.07	(0.76–1.5)	1.15	(0.76–1.73)
**Age**					
18–29	53	Ref		Ref	
30–39	56	1.12	(0.76–1.65)	1.26	(0.79–2.00)
40–49	49	0.85	(0.50–1.44)	1.22	(0.65–2.29)
>50	41	0.61	(0.33–1.11)	1.18	(0.56–2.46)
**Education**					
Less than high school	58	Ref		Ref	
High school graduate	53	0.82	(0.34–1.95)	0.87	(0.32–2.39)
Some college	44	0.56	(0.23–1.38)	0.54	(0.18–1.56)
College degree or higher	55	0.88	(0.38–2.04)	0.75	(0.26–2.15)
**Household income**					
Less than 8000 MXN monthly	53	Ref		Ref	
8001 to 15,000 MXN monthly	55	1.08	(0.65–1.79)	0.88	(0.48–1.60)
15,001 to 20,000 MXN monthly	55	1.10	(0.65–1.85)	0.96	(0.50–1.85)
>20,000 MXN monthly	50	0.89	(0.56–1.42)	0.85	(0.45–1.61)
Don’t know	63	1.50	(0.34–6.58)	4.55	(0.78–26.5)
**Data collection**					
Wave 1	52	Ref		Ref	
Wave 2	55	1.15	(0.78–1.71)	1.25	(0.78–1.98)
Wave 3	52	1.02	(0.66–1.55)	1.03	(0.62–1.69)
**Tobacco product use**					
Exclusive conventional smoker	39	Ref		Ref	
Dual sporadic user	61	**2.43 ^3^**	(1.63–3.62)	1.35	(0.82–2.23)
Dual frequent user	62	**2.52 ^3^**	(1.63–3.91)	1.58	(0.91–2.74)
**Cigaretteconsumption**					
Non-daily	49	Ref		Ref	
Daily ≤ 5 cigarettes	57	1.38	(0.89–2.13)	1.17	(0.70–1.96)
Daily > 5 cigarettes	55	1.29	(0.87–1.91)	0.98	(0.61–1.58)
**Flavor capsule use**					
No	40	Ref		Ref	
Yes	59	**2.13 ^3^**	(1.48–3.07)	1.35	(0.88–2.09)
**Recent quit attempt**					
No	46	Ref		Ref	
Yes	59	**1.69 ^2^**	(1.21–2.38)	**1.80 ^2^**	(1.17–2.77)
**Plan to quit**					
Sometime in the future	53	Ref		Ref	
In the next six months	52	0.94	(0.67–1.31)	**0.57 ^1^**	(0.37–0.89)
**E-cigarette ads on Internet**					
No	35	Ref		Ref	
Yes	60	**2.86 ^3^**	(1.95–4.18)	**1.98 ^2^**	(1.32–3.11)
**E-cigarette ads by email**					
No	43	Ref		Ref	
Yes	69	**3.02 ^3^**	(2.10–4.35)	**1.90 ^2^**	(1.23–2.92)
**Binge drinking**					
No	51	Ref		Ref	
Yes	57	1.32	(0.91–1.90)	0.95	(0.61–1.47)
**Marijuana use in the last month**					
None	44	Ref		Ref	
Once	60	**1.88 ^2^**	(1.16–3.05)	1.25	(0.71–2.21)
More than once	72	**3.17 ^3^**	(2.02–4.98)	**1.87 ^1^**	(1.11–3.15)
**Friends smokes**					
No	53	Ref		Ref	
Yes	53	0.97	(0.53–1.79)	**0.47 ^1^**	(0.23–0.99)
**Friends use e-cigarettes**					
No	35	Ref		Ref	
Yes	62	**3.08 ^3^**	(2.14–4.44)	**1.83 ^1^**	(1.11–3.01)
**Family smokes**					
No	41	Ref		Ref	
Yes	57	**1.88 ^3^**	(1.28–2.74)	1.36	(0.84–2.20)
**Family uses e-cigarettes**					
No	42	Ref		Ref	
Yes	67	**2.72 ^3^**	(1.91–3.86)	1.25	(0.77–2.02)

Significant values in bold: ^1^ *p*-value, *p* < 0.05; ^2^ *p*-value, *p* < 0.01; ^3^ *p*-value, *p* < 0.001.

**Table 4:** Factors associated with the interest in trying IQOS among adult smokers (*n* = 3219).

Variable	%	Univariate Estimates		Adjusted Estimates	
		OR	(95% CI)	AOR	(95% CI)
**Awareness**					
No	75	Ref		Ref	
Yes	77	1.15	(0.92–1.42)	0.79	(0.62–1.02)
**Gender**					
Female	74	Ref		Ref	
Male	76	1.07	(0.91–1.26)	0.97	(0.81–1.16)
**Age**					
18–29	74	Ref		Ref	
30–39	78	**1.26 ^1^**	(1.03–1.54)	1.18	(0.95–1.47)
40–49	78	1.25	(0.98–1.58)	1.26	(0.97–1.63)
>50	70	0.84	(0.67–1.05)	0.97	(0.75–1.26)
**Education**					
Less than high school	67	Ref		Ref	
High school graduate	73	1.29	(0.98–1.70)	1.18	(0.88–1.58)
Some college	75	**1.42 ^1^**	(1.05–1.91)	1.21	(0.88–1.67)
College degree or higher	80	**1.89 ^3^**	(1.42–2.51)	1.28	(0.93–1.77)
**Household income**					
Less than 8000 MXN monthly	69	Ref		Ref	
8001 to 15,000 MXN monthly	75	**1.35 ^2^**	(1.09–1.68)	1.17	(0.94–1.48)
15,001 to 20,000 MXN monthly	77	**1.44 ^2^**	(1.12–1.85)	1.09	(0.83–1.44)
>20,000 MXN at monthly	81	**1.86 ^3^**	(1.47–2.34)	**1.37 ^1^**	(1.05–1.79)
Don’t know	64	0.77	(0.54–1.10)	0.83	(0.57–1.21)
**Data collection**					
Wave 1	74	Ref		Ref	
Wave 2	76	1.11	(0.92–1.34)	1.20	(0.99–1.46)
Wave 3	74	0.98	(0.80–1.20)	1.05	(0.85–1.29)
**Tobacco product use**					
Exclusive conventional smoker	71	Ref		Ref	
Dual sporadic user	80	**1.70 ^3^**	(1.38–2.09)	**1.42 ^2^**	(1.10–1.82)
Dual frequent user	87	**2.67 ^3^**	(1.99–3.59)	**1.90 ^3^**	(1.37–2.64)
**Cigarette consumption**					
Non-daily	71	Ref		Ref	
Daily ≤ 5 cigarettes	77	**1.38 ^2^**	(1.12–1.69)	**1.41 ^2^**	(1.13–1.74)
Daily > 5 cigarettes	81	**1.78 ^3^**	(1.44–2.19)	**1.79 ^3^**	(1.42–2.25)
**Flavor capsule use**					
No	73	Ref		Ref	
Yes	76	**1.19 ^1^**	(1.02–1.40)	1.19	(1.00–1.42)
**Recent quit attempt**					
No	73	Ref		Ref	
Yes	77	**1.23 ^1^**	(1.05–1.45)	**1.22 ^1^**	(1.01–1.48)
**Plan to quit**					
Sometime in the future	75	Ref		Ref	
In the next six months	76	1.07	(0.90–1.26)	0.95	(0.78–1.15)
**E-cigarette ads on Internet**					
No	70	Ref		Ref	
Yes	81	**1.84 ^3^**	(1.56–2.17)	**1.77 ^3^**	(1.47–2.15)
**E-cigarette ads by email**					
No	75	Ref		Ref	
Yes	75	0.97	(0.78–1.22)	**0.62 ^3^**	(0.48–0.81)
**Binge drinking**					
No	73	Ref		Ref	
Yes	82	**1.76 ^3^**	(1.43–2.17)	**1.54 ^3^**	(1.24–1.92)
**Marijuana use in the last month**					
None	75	Ref		Ref	
Once	70	**0.76 ^1^**	(0.59–0.98)	**0.65 ^2^**	(0.50–0.86)
More than once	78	1.16	(0.90–1.49)	0.89	(0.67–1.18)
**Friends smokes**					
No	68	Ref		Ref	
Yes	76	**1.52 ^3^**	(1.24–1.87)	1.21	(0.97–1.50)
**Friends use e-cigarettes**					
No	71	Ref		Ref	
Yes	82	**1.81 ^3^**	(1.52–2.15)	**1.35 ^2^**	(1.08–1.68)
**Family smokes**					
No	75	Ref		Ref	
Yes	75	1.00	(0.85–1.18)	0.99	(0.82–1.18)
**Family uses e-cigarettes**					
No	74	Ref		Ref	
Yes	78	**1.23 ^1^**	(1.02–1.50)	0.82	(0.64–1.04)

Significant values in bold: ^1^ *p*-value, *p* < 0.05 ^2^ *p*-value, *p* < 0.01; ^3^ *p*-value, *p* < 0.001.

## Supplementary Materials

The following are available online at https://www.mdpi.com/1660-4601/17/7/2173/s1, Table S1: Factors associated with the interest in trying IQOS, among adult smokers who were aware of HTPs (*n* = 550).
