# Supplementary material for: Adult Smokers’ Awareness and Interest in Trying Heated Tobacco Products: Perspectives from Mexico, where HTPs and E-Cigarettes are Banned
Source: Int J Environ Res Public Health. 2020 Mar 25;17(7):2173. doi: 10.3390/ijerph17072173 (PMC7177513; doi:10.3390/ijerph17072173)
Supplement: Supplementary file 1 [file ijerph-17-02173-s001.pdf]

Supplementary Material

Table S1. Factors associated with the interest in trying IQOS, among adult smokers who were aware of HTPs (n = 550).

Variable		Univariate estimates		Adjusted estimates	
	%	OR	(95% CI)	AOR	(95% CI)
Gender					
Female	77	Ref		Ref	
Male	78	1.06	(0.71–1.58)	1.12	(0.70–1.78)
Age					
18–29	72	Ref		Ref	
30–39	81	1.62 [1]	(1.02–2.56)	1.46	(0.86–2.48)
40–49	80	1.49	(0.79–2.82)	1.05	(0.50–2.18)
>50	73	1.04	(0.53–2.03)	0.85	(0.38–1.93)
Education					
Less than high school	88	Ref		Ref	
High school graduate	78	0.51	(0.14–1.82)	0.29	(0.07–1.13)
Some college	76	0.44	(0.12–1.61)	0.21 [1]	(0.05–0.86)
College degree or higher	76	0.45	(0.13–1.57)	0.14 [2]	(0.03–0.56
Household income					
Less than 8000 MXN monthly	68	Ref		Ref	
8001 to 15,000 MXN monthly	73	1.28	(0.74–2.22)	1.04	(0.5–1.96)
15,001 to 20,000 MXN monthly	85	2.6 [2]	(1.39–5.08)	2.7 [2]	(1.29–5.86)
>20,000 MXN monthly	82	2.1 [2]	(1.24–3.66)	2.2 [1]	(1.12–4.64)
Don't know	75	1.44	(0.28–7.49)	2.67	(0.46–15.36)
Data collection					
Wave 1	74	Ref		Ref	
Wave 2	81	1.48	(0.92–2.40)	1.51	(0.89–2.57)
Wave 3	80	1.48	(0.80–2.49)	1.19	(0.67–2.14)
Tobacco product use					
Exclusive conventional smoker	71	Ref		Ref	
Dual sporadic user	76	1.27	(0.82–1.98)	1.43	(0.81–2.51)
Dual frequent user	88	3.0 [3]	(1.69–5.49)	3.0 [2]	(1.53–6.20)
Cigarette consumption					
Non-daily	75	Ref		Ref	
Daily ≤5 cigarettes	77	1.11	(0.67–1.85)	0.76	(0.42–1.35)
Daily >5 cigarettes	81	1.40	(0.87–2.26)	0.98	(0.56–1.71)
Flavor capsule use					
No	75	Ref		Ref	
Yes	78	1.20	(0.79–1.83)	1.27	(0.76–2.10)
Recent quit attempt					
No	74	Ref		Ref	
Yes	80	1.43	(0.96–2.14)	1.67 [1]	(1.01–2.73)
Plan to quit					
Sometime in the future	79	Ref		Ref	
In the next six months	75	0.78	(0.53–1.17)	0.72	(0.44–1.18)
E-cigarette ads on Internet					
No	68	Ref		Ref	
Yes	81	1.9 [2]	(1.29–2.97)	2.0 [2]	(1.25–3.28)
E-cigarette ads by email					
No	78	Ref		Ref	
Yes	75	0.83	(0.55–1.24)	0.74	(0.45–1.24)
Binge drinking					
No	74	Ref		Ref	
Yes	83	1.7 [1]	(1.07–2.72)	1.44	(0.85–2.44)
Marijuana use in the last month					
None	81	Ref		Ref	

Once	68	0.5 [1]	(0.31–0.88)	0.55	(0.29–1.03)
More than once	73	0.66	(0.41–1.08)	0.49[1]	(0.27–0.88)
Friends smokes					
No	69	Ref		Ref	
Yes	78	1.58	(0.81–3.08)	1.27	(0.58–2.80
Friends use e–cigarettes					
No	73	Ref		Ref	
Yes	79	1.42	(0.95–2.14)	1.52	(0.85–2.72)
Family smokes					
No	74	Ref		Ref	
Yes	78	1.30	(0.84–2.00)	1.37	(0.79–2.36)
Family uses e–cigarettes					
No	78	Ref		Ref	
Yes	77	0.94	(0.63–1.41)	0.58	(0.32–1.04)

[1]p-value, $p < 0.05$; [2]p-value, $p < 0.01$; [2]p-value, $p < 0.01$; and [3]p-value; $p < 0.001$.
